# AI-Driven vision large language framework for pediatric pain assessment

**DOI:** 10.3389/fped.2026.1784197

**Published:** 2026-07-22

**Authors:** Bing Li, Jinguo Pang, Yaxin Cai, Jing Yu, Yong Liu, Renrui Tian, Peng Yu, Xiaoling Liang, Shiqing Wang, Peiyuan Wu, Lulu Yang, Lingxiao Wu, Zengxiao Zhao, Li Zheng, Kangning Zhang, Liping Wang, Li Yang, Laurent Itti, Shixian Wen

**Affiliations:** 1 Department of Anesthesiology, Department of Pediatrics, Women and Childrens Hospital, School of Medicine, Xiamen University, Xiamen, China; 2 Southern University of Science and Technology, Shenzhen, China; 3 School of Biomedical Engineering, Shenzhen University Medical School, Shenzhen University, Shenzhen, China; 4 Department of Behavioral Development, Guangzhou Children and Women Medical Center, Guangzhou Medical University, Guangzhou, China; 5 Guangdong Provincial Key Laboratory of Brain Connectome and Behavior, the Brain Cognition and Brain Disease Institute, Shenzhen Institutes of Advanced Technology, Chinese Academy of Sciences, Shenzhen, China; 6 Department of Computer Science and Technology, Tsinghua University, Beijing, China; 7 Department of Anesthesiology, Children’s Hospital Affiliated to Zhengzhou University, Zhengzhou, China; 8 China Academy of Information and Communications Technology, Beijing, China; 9 Brain Everest LLC, Shenzhen, China; 10 CAS Key Laboratory of Brain Connectome and Manipulation, Shenzhen-Hong Kong Institute of Brain Science, Shenzhen Institutes of Advanced Technology, Chinese Academy of Sciences, Shenzhen, China; 11 Thomas Lord Department of Computer Science, University of Southern California, Los Angeles, CA, United States

**Keywords:** FLACC scale, pediatric pain management, prompt engineering, real-world clinical dataset, vision large language framework

## Abstract

**Background:**

Accurate pediatric pain assessment is essential for effective pain management and procedural safety. However, current evaluations largely rely on subjective scales and casual behavioral observations. Although automated pain assessment methods have been proposed, they often remain complex and less reliable than expert clinicians. This study aimed to establish a proof-of-concept for a novel Pain Assessment Vision–Large Language Framework (PA-VLLF) for consistent pediatric pain evaluation.

**Methods:**

In this observational proof-of-concept study, we developed and validated the PA-VLLF using representative keyframes extracted from real-world surveillance video recordings of venipuncture from two camera angles. The foundation framework combined ChatGPT-4o and Qwen2-VL vision-language architectures, with customized prompts and fine-tuning to generate FLACC (Facial, Legs, Activity, Cry, Consolability) scores within a human-in-the-loop framework. Data were collected and analyzed from September to December 2024. The study was approved by the institutional review board (IRB no. 294A01) and parental consent was obtained.

**Results:**

We established a Clinical Pain Assessment (CPA) dataset of 1,248 video segments from 104 children, independently annotated by five certified pain experts over a cumulative 950 person-hours to establish a consensus baseline. In this dataset, PA-VLLF-ChatGPT successfully aligned with the collective expert consensus, achieving 86.36% accuracy on expert-labeled pain scores, performing comparably to senior experts (84.69%, *p* > 0.05) and outperforming machine-learning approaches in our comparisons; precision of pain-level assessment was evaluated using complementary metrics (MAX agreement and Z-Score).

**Conclusions:**

As a successful proof-of-concept, PA-VLLF demonstrates that vision-language models can successfully internalize expert-derived clinical reasoning. By serving as a standardized, consensus-based intelligent assistant, it reduces the cognitive burden of complex visual evaluations and mitigates individual rater variance. This framework holds promise for improving pain management strategies, supporting highly consistent, AI-assisted clinical decision-making. To support future research, the CPA dataset will be accessible upon the publication of this article to qualified researchers under data privacy regulations.

## 1 Introduction

Pain, recognized as the fifth vital sign, is as important as heart rate, blood pressure, respiratory rate, and oxygen saturation (1). Accurate assessment and effective management of pain are essential for improving patients' quality of life. Pain can originate from various sources. In addition to underlying medical conditions such as appendicitis, arthritis, and migraine, procedure-related pain—resulting from interventions like surgery, lumbar puncture, and venipuncture—can impose a substantial burden and lead to significant adverse effects (2). Among these, blood draws and venipuncture are among the most common procedures. As venipuncture is a routine clinical practice, nearly all hospitalized patients undergo at least one such procedure during their course of treatment (3).

Unlike adults, children tend to amplify the experience of pain due to limited cognitive development and vivid imagination. The impact of pain in pediatric patients extends beyond physical discomfort and often manifests more profoundly at the psychological level. Inadequate pain management can result in increased anxiety and may lead to long-term adverse outcomes that persist into adulthood (4, 5). For example, painful memories and fear associated with pain can lead to resistance during the procedure, which may then result in procedural failure and complications, including venous thrombosis, infection, and nerve damage (6–8). In the long run, pain during venipuncture can increase anxiety and resistance during subsequent medical visits, and reduce compliance with significant procedures such as vaccinations (9, 10). Therefore, accurate pain assessment then the implementation of effective interventions are particularly important for safeguarding the psychological well-being of pediatric patients during medical care. The most widely used pediatric pain assessment scale in clinical practice is the FLACC scale (Face, Legs, Activity, Cry, Consolability) (11, 12). It offers a standardized approach to clinical pain assessment and serves as an effective tool for assessing pain in children who cannot clearly verbalize pain. However, the FLACC scale is based on observations that are not always quantifiable or specific (e.g., whether the patient frowned or cried). As a result, the interpretation of these observations can vary among different raters, leading to subjective and inaccurate scoring outcomes (13).

These challenges raise the question of whether it is possible to consistently and accurately assess venipuncture pain in real clinical scenarios. A wide range of pain assessment methods have been develope (14–17), although their precision is below that of the expert clinician. Furthermore, many of these methods rely on facial analysis videos that require well-framed close-up shots of the face (15, 18). They may be limited by the availability and quality of the data (19, 20)^,^ and also often depend on additional equipment (15, 21) to monitor vital signs (e.g., electroencephalogram EEG, electrodermal activity EDA, electromyography EMG, and electrocardiogram ECG). This complicates their implementation in some clinical environments. In light of these limitations, we wonder whether the use of machine vision and artificial intelligence technology could improve the accuracy of estimation in varied real-world clinical scenarios, simply by analyzing video and audio footage from surveillance cameras already present in the procedure room.

The development of a standardized, AI-assisted, and noncontact pain assessment system is of critical importance. In pediatric venipuncture, prolonged pain can increase a child's fear, triggering resistance that can compromise procedural success. In contrast, rapid and accurate pain assessment allows nurses to quickly implement analgesic interventions, such as therapeutic massage, pain relief aids such as Buzzy (Vibrating Ice Pack) (22), or topical anesthetics to alleviate discomfort. This approach not only improves the child's experience during venipuncture, but also mitigates long-term medical anxiety. By integrating artificial intelligence and machine vision technology, healthcare providers could minimize evaluator bias while ensuring timely pain management. This innovation holds the potential to improve clinical workflows, ensuring a better experience that benefits both immediate procedural success and long-term psychological well-being.

## 2 Related work

Numerous research efforts have been made to estimate the pain level in humans utilizing videos. Earlier deep learning methods extensively relied on Convolutional Neural Networks (CNNs) (23) and Long Short-term Memory (LSTM) (24) networks to capture the spatial and dynamic nature of facial expressions. Recent advances have seen a shift towards transformer-based architectures and foundation models to capture more complex feature representations. A full transformer-based framework incorporating a Transformer-in-Transformer (TNT) module for spatial feature extraction, along with a temporal transformer leveraging both cross-attention and self-attention mechanisms, has been proposed to model spatiotemporal dynamics more effectively (25). To integrate multimodal information without relying on physical thermal sensors, subsequent work employed Generative Adversarial Networks (GANs) to synthesize thermal video data from RGB inputs, which were then processed through a Vision-MLP and transformer-based pipeline (26). More recently, PainFormer was introduced as a vision foundation model trained via large-scale multi-task learning. Designed as a universal embedding extractor, it enables the integration of diverse behavioral and physiological modalities, including RGB, depth, synthetic thermal videos, and multiple biosignals, thereby substantially enhancing model generalizability (27).

Despite the impressive state-of-the-art performances of these models, the majority of these studies are validated on adult datasets under strictly controlled laboratory conditions (e.g., experimentally induced heat pain). Assessing pediatric procedure-related pain, such as venipuncture, in real-world clinical scenarios presents unique challenges, including unconstrained body movements, parental soothing, and occlusions. Furthermore, clinical environments require highly interpretable outputs rather than abstract pain intensity scores. To address these gaps, our study establishes a proof-of-concept that leverages Vision Large Language Frameworks (VLLFs) guided by prompt engineering to directly align AI-assisted pain recognition with the clinically established FLACC scale.

## 3 Materials and methods

### 3.1 Study design and ethical approval

This diagnostic study was approved by the Ethics Committee of Guangzhou Women and Children's Medical Center, Guangzhou Medical University (GWCMC). All procedures were conducted in accordance with the Declaration of Helsinki.

### 3.2 Participants

**Figure 1:**
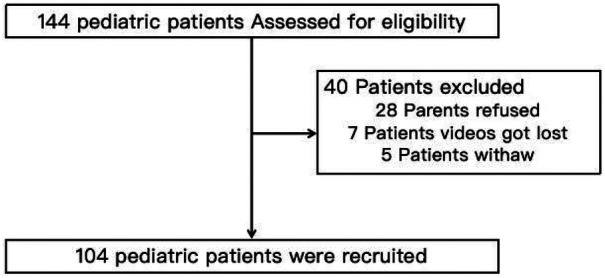
Participant recruitment flowchat.

This study was conducted at the Outpatient Department of Children's Health Checkup, Guangzhou Women and Children's Medical Center from April 2024 to September 2024. Venipuncture for patients under 12 years old was performed by an experienced nurse in a designated treatment room. Inclusion criteria were: (1) age under 12 years, (2) scheduled for venipuncture. Exclusion criteria included: (1) known pain perception impairments; (2) a history of chronic pain, seizures or motion sickness. All eligible participants were recruited after obtaining written consent (Figure 1), permitting secondary analysis of anonymized and securely stored data.

### 3.3 Data acquisition

**Figure 2:**
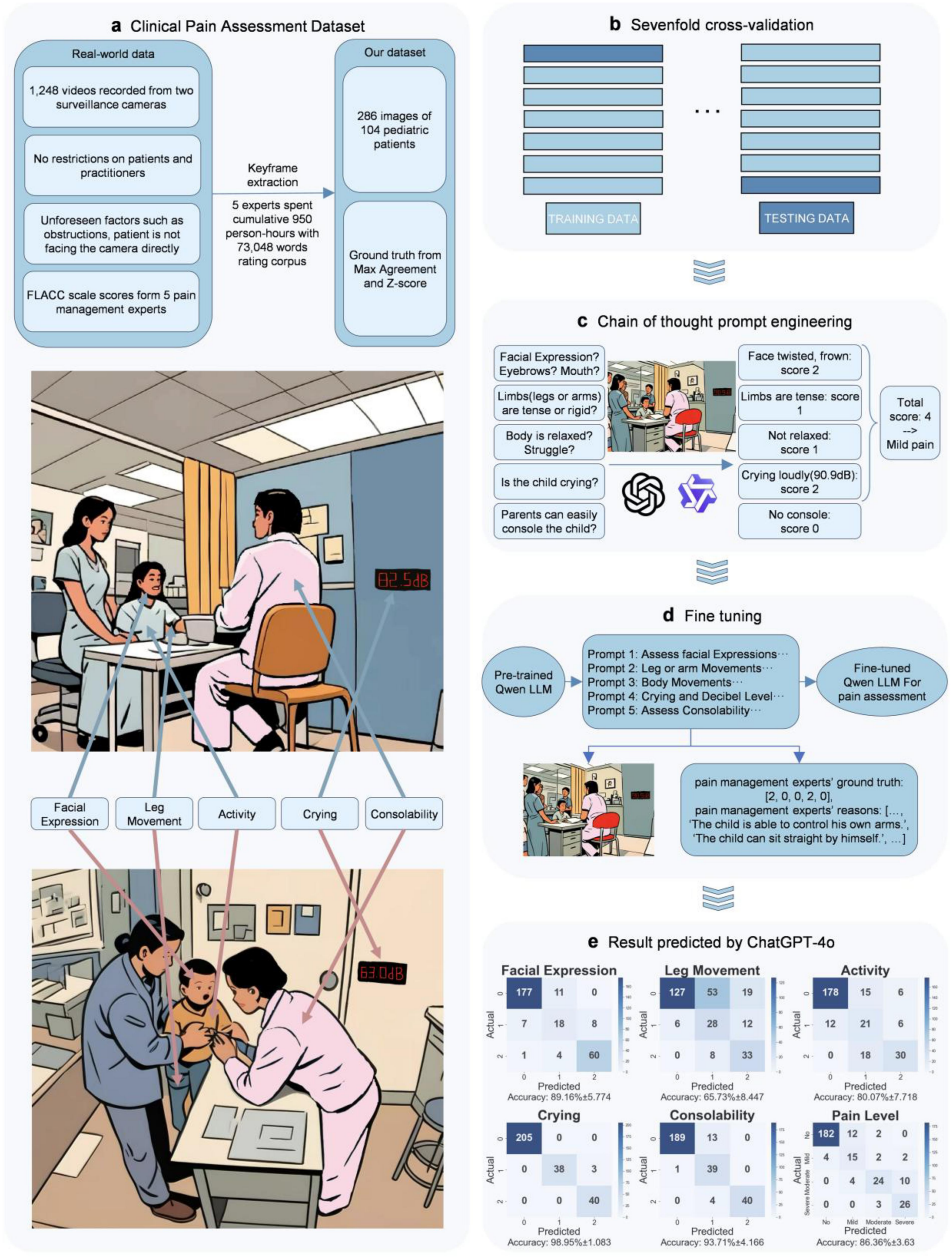
Pain assessment-vision large language framework. **(a)** CPA dataset architecture and representative video frame pairs captured from two surveillance cameras (The images shown are AI-generated illustrations based on real clinical video frames to protect the privacy of patients and medical staff), accompanied by their corresponding FLACC scale annotations. **(b)** A 7-fold cross-validation protocol was implemented during model development. **(c)** Chain-of-Thought (CoT) Prompt engineering. **(d)** Domain-specific fine-tuning of PA-VLLF with detailed rating corpus. **(e)** Confusion matrix of pain assessment results by PA-VLLF-ChatGPT4o.

We used two different angled surveillance cameras to capture the scenarios of pediatric patients undergoing venipuncture in a real clinical setting without restrictions for patients and practitioners (Figure 2a). Video segments spanning from the initiation of blood collection (skin disinfection) to procedural completion (cotton ball pressure for hemostasis) were selected. Technicians extracted corresponding images and audio components from these segments as a Clinical Pain Assessment (CPA) Dataset to enable pain assessment and scoring. Five pain assessment experts established the ground truth by evaluating extracted image and audio data, based on the FLACC (Face, Legs, Activity, Cry, Consolability) scale criteria (Supplementary Table S1).

### 3.4 CPA dataset and annotations

The CPA dataset was derived from a total of 6,240 min of continuous raw surveillance video recordings collected from 104 patients. From this extensive raw footage, we extracted 1,248 specific venipuncture video segments (each 10 s in duration, totaling 208 min). From these recordings, 286 high-quality keyframes were manually selected for pain assessment. Since a video is fundamentally a sequence of overlapping image frames, and venipuncture induces a highly acute, short-lived pain response, extracting the most representative image effectively captures the peak pain expression. Therefore, using the most representative static keyframes serves as a scientifically valid and highly efficient proxy for this proof-of-concept study. The selection ensured clear visibility of the patient with minimal or no occlusion. All images were captured in real-world clinical settings using a dual-camera surveillance system. To minimize visual obstruction from medical staff or caregivers and to comprehensively capture both facial expressions and body movements required for FLACC scoring, the cameras were strategically positioned at the patient's front-left (approximately +45° to the sagittal plane) and front-right (approximately −45°). The 286 keyframes represent 286 independent evaluation instances. In real-world clinical settings, occlusions by medical staff are frequent. The dual-camera setup maximizes the probability of capturing a clear view. Furthermore, to maximize data utilization and justify the high costs of clinical data acquisition, multiple distinct keyframes were extracted from the same patient if they presented clear, valid pain expressions. Since the PA-VLLF processes each image entirely independently without temporal memory, treating each keyframe as an individual inference sample is standard and appropriate for evaluating the algorithm's image-level recognition capability. Each image was manually annotated with the patient's crying state: no crying, occasional crying, or continuous crying based on the corresponding audio track, serving as a prompt for experts and PA-VLLF scoring. Five pain management experts dedicated 950 person-hours to scoring these images based on the FLACC scale (Supplementary Table S1). They also compiled a comprehensive rating corpus (73,048 words) for each patient, documenting the rationale behind their score assignments. Detailed pain expert scoring guidelines are provided in Supplementary Figures S1–S5.

To justify the adequacy of our curated dataset size, an a-priori power analysis was conducted using G*Power 3.1. Because the primary objective of this study is to validate the computer vision model's classification accuracy, the statistical unit of analysis for evaluating model performance is the individual representative keyframe rather than the patient. Approaching the 4-class pain level evaluation across these keyframes, we set a medium effect size (f = 0.25), a significance level (*α*= 0.05), and a stringent statistical power (1 − *β* = 0.95). The analysis indicated a minimum requirement of 280 evaluating instances. Our highly curated benchmark subset comprises 286 representative keyframes, successfully exceeding this threshold. This ensures sufficient statistical power at the image-level to robustly distinguish between the four pain categories.

### 3.5 Model development

The input of PA-VLLF was CPA data with our custom prompt based on FLACC score criteria, the algorithm was asked to generate FLACC scores. Due to ethical reasons, we did not fine-tune ChatGPT-4o (28) on images of faces or children. We selected the Qwen2-VL (29) model for fine-tuning, available in 7B and 72B versions.

#### 3.5.1 Prompt engineering

We crafted a set of zero-shot chain-of-thought prompts (30) for pain assessment, consisting of a component system (Supplementary Figures S1–S5) and a component “user” (Supplementary Figures S1–S5). The prompt of the system component incorporated chain-of-thought and reflection methodologies, guiding the model to deliver a detailed reasoning process and self-reflection during problem solving. The “user” dimension's prompt is based on the FLACC scale, providing specific instructions for task completion. Divide the task into five dimensions: Face, Legs, Activity, Cry, and Consolability. The prompt of each component further broke down the subquestions into multiple specific steps.

To enhance the accuracy of the assessment, we enforced the large language model to output in a specific format, which involves providing the basis before assigning a score. This was also part of the chain-of-thought and improved the interpretability of the results. Large language models are sensitive to exact prompts. Through manual design and adjustment of chain-of-thought prompts, we achieved better performance compared to direct prompts.

#### 3.5.2 Fine-Tuning

We employed LLaMA Factory (31) for fine-tuning, which encompassed both the fine-tuning dataset preparation and the Supervised Fine-Tuning (SFT) stages. In the fine-tuning dataset preparation phase, we constructed our data set referencing the Alpaca format for multimodal datasets. During the SFT stage, we leveraged DeepSpeed ZeRO-3 and LowRank Adaptation (LoRA) (30) to alleviate GPU memory consumption. Furthermore, the corresponding pseudocode was provided in the Supplementary Algorithms (Supplementary Figure S6, Table S2).

We employed a 7-fold cross-validation based on the seven separate days of data collection. Since patients did not overlap across days, this strategy ensures patient-level stratification and prevents data leakage between training and testing sets. The training hyperparameters were optimized independently for the 7B and 72B models (Supplementary Table S3). The loss curves for the training and evaluation sets of the 7B and 72B models are presented in Supplementary Figure S6.

We merged the adapter corresponding to the step with the lowest evaluation loss.

The fine-tuning process was performed using torchrun for distributed training on two NVIDIA A800 GPUs. Under these configurations, fine-tuning the 7B model required approximately 40 min per run, whereas the 72B model took around 3 h per run.

### 3.6 Statistical analysis

We compared the precision of pain level assessment of PA-VLLF with pain assessment experts using two complementary metrics: MAX agreement (32) and Z-Score (33).

#### 3.6.1 MAX agreement

Determines consensus by majority voting among expert evaluations. For each image and FLACC scale feature, five pain management experts initially provided independent scores S1, S2, S3, S4, S5. The ground truth was established as follows:

Ground Truth={mode(S1,S2,S3,S4,S5)if ∃s∈{Si} with ∑i=15 I(Si=s)≥3Expert consensus through discussionotherwise

where I (·) denotes the indicator function. Images without majority agreement were flagged as **UNCERTAIN** and resolved through a panel discussion, replicating clinical consultation workflows. The precision of the final model was calculated as the percentage of predictions that matched the established ground truths in all images.

#### 3.6.2 Z-Score

Measures deviation from expert consensus distribution. For each image and FLACC feature, we first calculated the mean (µ) and the population standard deviation (σ) of the expert scores:

$$\mu =\frac{1}{5}\overset{5}{\underset{i=1}{\sum }}\;S_{i}$$

$$\sigma =\sqrt{\frac{1}{5}\overset{5}{\underset{i=1}{\sum }}\;{\left(S_{i}-\mu \right)}^{2}}$$

The Z scores for both expert ratings and PA-VLLF predictions were computed as:

$$Z=\frac{S-\mu }{\sigma }$$

where S represents any score (expert or model).

The model predictions were considered to be consistent with the expert consensus if

$$|Z_{\mathrm{model}}|<\frac{1}{5}\overset{5}{\underset{i=1}{\sum }}\;|Z_{\mathrm{exper}t_{i}}|$$

This zero-threshold criterion ensures that model predictions fall below the expert mean, reflecting conservative pain assessment aligned with clinical safety priorities. The precision of the final Z score represents the proportion of predictions that meet this condition in all assessments.

#### 3.6.3 Baselines

We compared the precision of pain level assessment of PA-VLLF with other baseline models. We employed three widely recognized models as baselines: SVM8, ResNet-5019, and the Children's Pain Assessment Neural Network (CPANN) 9. Each model was carefully configured to suit the specific characteristics of our dataset. (Supplementary Tables S4, S5).

#### 3.6.4 SVM

SVM finds an optimal hyperplane that maximizes the margin between classes for efficient classification or regression. It is widely used in text classification, face recognition, and anomaly detection.

#### 3.6.5 ResNet-50

ResNet-50 (34) is a deep convolutional neural network that uses residual learning with skip connections to mitigate vanishing gradients, enabling efficient training of very deep networks.

#### 3.6.6 CPANN-based method

CPANN is a deep learning model designed to assess postoperative pain in children. It operates by extracting spatial information from video frames using a CNN (Convolutional Neural Network) backbone. Subsequently, it enhances the representation of pain features and diminishes the influence of irrelevant noise through the Semantic Attention Module (SAM) and the Differential Attention Module (DAM). Given the absence of preoperative data in our data set, we adapt the CPANN architecture by excluding modules designed for preoperative image processing and noise reduction. Specifically, we retained the Semantic Attention Module (SAM) to focus on pain-related features while omitting the Disparity Attention Module (DAM).

## 4 Results

### 4.1 CPA dataset characteristics

Our CPA data set was collected at the Outpatient Department of Children's Health Checkup of Guangzhou Women and Children's Medical Center. It includes 104 pediatric patients who received venipuncture to obtain blood collection (Table 1). Needle-related procedures induce acute pain, and none of the children in our data set had a history of chronic pain before undergoing venipuncture. Patients include preschool- and school-age children, with age distributions of under 36 months (45.2%), 37–72 months (38.5%), and over 72 months (16.3%), and a male-to-female ratio of approximately 70:30. We obtained a total of 6,240 min of continuous raw surveillance video recordings, captured from two cameras in a real clinical setting without restrictions for patients and practitioners. From this extensive raw footage, we extracted 1,248 specific venipuncture video segments (each 10 s in duration, totaling 208 min). Subsequently, we extracted a curated benchmark subset of 286 representative images across the 104 pediatric patients to reflect the full spectrum of both pain and no-pain conditions. Five experienced pain management experts initially assessed the pain scores of these images in an independent manner using the FLACC scale (Face, Leg, Activity, Cry, and consolability). Importantly, because the dataset was derived from real-world clinical video recordings with complex environmental noise, clean isolated audio could not be reliably extracted. Therefore, the Cry scores were manually pre-annotated and provided as a known contextual variable. The experts then independently evaluated the remaining four visual dimensions. They spent a cumulative of 950 person-hours on pain assessments and wrote a corpus of 73,048 words rating (Figure 2a).

**Table 1:** Pediatric patients' demographics and diagnosis.

Pediatrics Patients (*n* = 104)	
Characteristics	*n* (%)
Age in months (%)	
≤36	47 (45.2%)
37–72	40 (38.5%)
>72	17 (16.3%)
Sex (%)	
Boys	73 (70.2%)
Girls	31 (29.8%)
Region (%)	
Guangzhou City	72 (69.2%)
Other Cities	32 (30.8%)

We established the ground truth metrics for each image using two metrics (Method Metrics): 1. Max Agreement Metric: mode of score evaluated by five pain management experts. 2. Z Score Metric: how many standard deviations a data point has from the mean scores evaluated by five pain management experts.

### 4.2 Expert-level pain assessment

**Figure 3:**
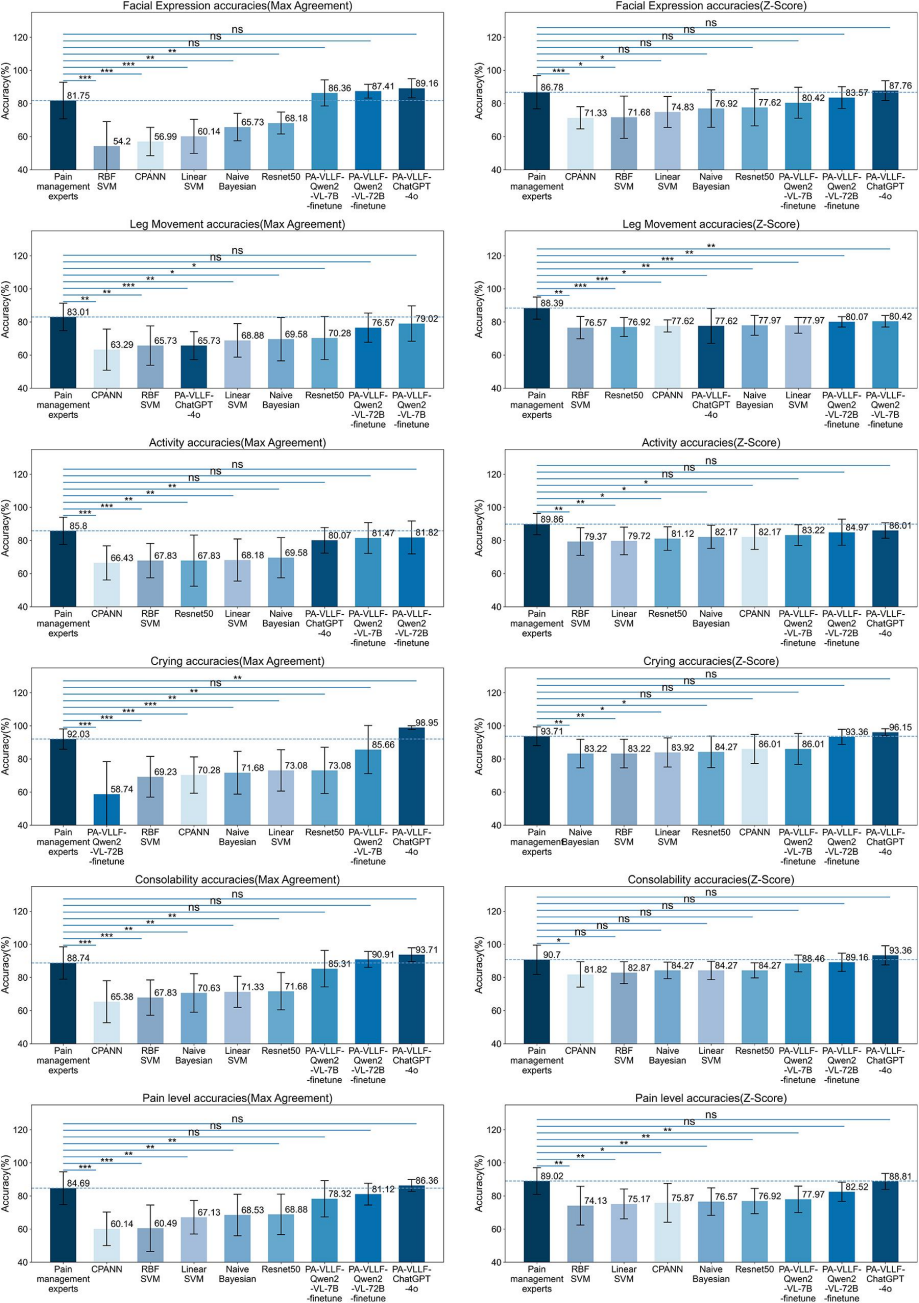
Comparison between PA-VLLF and baselines.

PA-VLLF demonstrated expert-level performance in pain assessment, successfully aligning with the collective expert consensus, surpassing other machine learning models in the CPA data set, as measured by the Max Agreement metrics (Figure 3a) and the Z Score metrics (Figure 3b). Specifically, the PA-VLLF-ChatGPT and PA-VLLF-Qwen-72B refinement achieved an accuracy of 86.36% and 84.97% in Max Agreement. Notably, PA-VLLF-ChatGPT performed comparably to senior pain management experts (86.36% vs. 84.69%, *p* > 0.05, ns), and significantly outperformed baseline models including CPANN 9, Support Vector Machine (SVM) 8, SVM with Radial Basis Function (RBF SVM) 8, ResNet5019, Qwen2-VL-7B-finetune and Qwen2-VL-72B-finetune by 1.67%, 26.22%, 19.23%, 25.87%, 17.48%, 8.04% and 5.24%, respectively. PA-VLLF-Qwen-finetune demonstrated comparable gains. In the Z score metric, the PA-VLLF-ChatGPT and PA-VLLF-Qwen-72B refinement achieved 88.81% and 82.52%, outperforming the other baseline models by 14.68% to 10.84%.

PA-VLLF, functioning as an intelligent assistant, also showed expert-level precision in all five FLACC dimensions (Figure 3), providing precise guidance to optimize venipuncture procedures. For example, PA-VLLF-ChatGPT achieved accuracies of 89.16% for Face, 98.95% for Cry and 93.71% for Consolability. Importantly, the near-perfect performance on the Cry dimension reflects the controlled contextual baseline provided equally to both the model and human raters, a methodological design necessitated by the lack of clean, isolated audio in real-world clinical recordings. Consequently, the model's independent visual recognition capabilities are most accurately demonstrated by its robust performance across the remaining purely visual dimensions. Across these visual domains, the results are on par with the performance of pain management experts (no statistically significant differences, as indicated by “ns” in Figure 3), while significantly outperforming baseline models by up to 32.17%, 28.67%, and 28.33% respectively. Furthermore, we implemented the Naive Bayes model as most children in real scenarios showed no obvious pain. This model assumes that all the data have no pain. In pain level assessment, PA—VLLF outperformed the Naive Bayes model by 17.83% (Max Agreement metric) and 12.24% (Z—Score metric), demonstrating the superiority of our model.

In our data, “no pain” constitutes the majority at 68.53%, a proportion that is consistent with the state of nature. This imbalance results in the Naive Bayes model outperforming the CPANN, RBF SVM, and Linear SVM by 8. 39%, 8. 04% and 1. 4% (Max Agreement) and 0.7%, 2.44%, 1.4% (Z-Score), respectively. However, in the evaluation of crying, CPANN outperforms Naive Bayes by 2. 79% (Z-Score) and Linear SVM outperforms Naive Bayes by 1.4% (Max Agreement) and 0.7% (Z-Score). In the consolability assessment, linear SVM outperforms naive Bayes by 0. 7% (Max Agreement), demonstrating that these baseline models are indeed effective.

### 4.3 Ambiguity reduction and confusion matrices

**Figure 4:**
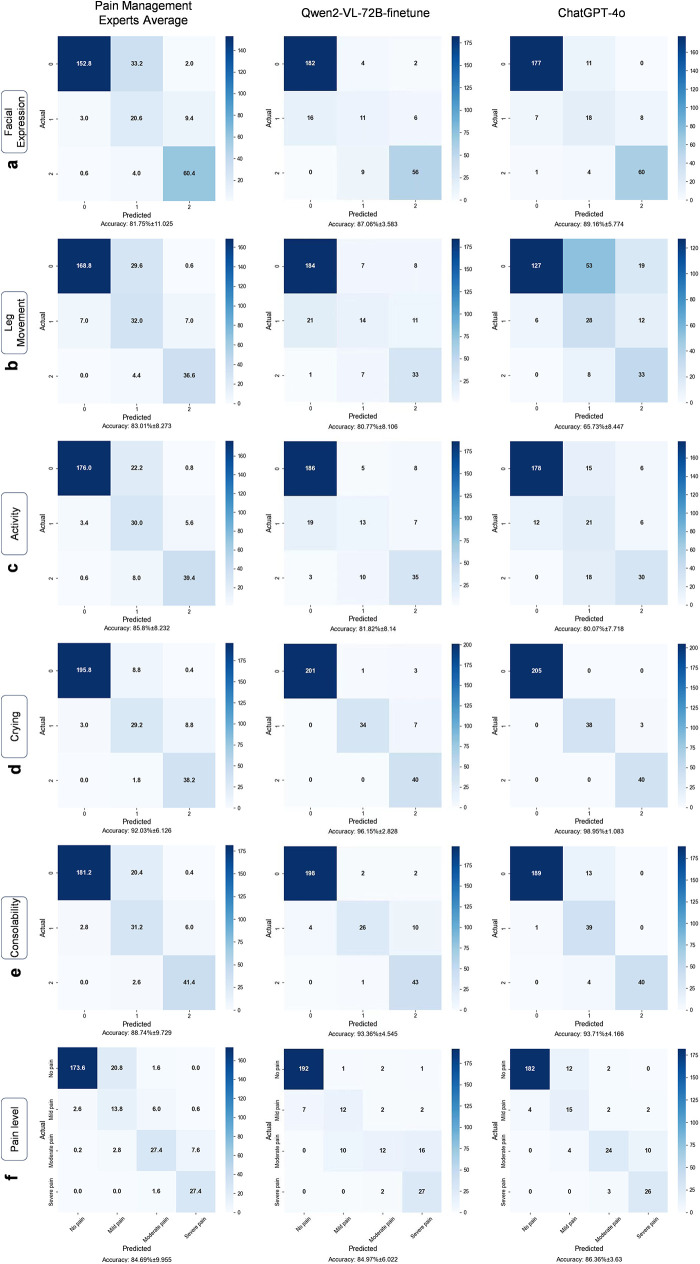
Confusion matrices of pain assessment results by pain management experts and PA-VLLF **(a)** facial expression, **(b)** leg movement, **(c)** activity, **(d)** crying, **(e)** consolability, and **(f)** resulting compound pain level.

PA-VLLF exhibited a robust discriminative capacity in resolving ambiguous scores ranging from no pain to mild pain. The confusion matrix analysis (Figure 4) demonstrated a high consistency in classification accuracy, with PA-VLLF showing a strict adherence to the established expert consensus when discriminating between the 0 and 1 sore levels (Figures 4a–e).

In the total pain level score (Figure 4f), the PA-VLLF-Qwen-72B-finetune and ChatGPT-4o variants identified 192 and 182 instances of no pain, respectively, compared to the expert average of 173.6. Rather than indicating a superhuman objectivity, these results demonstrate that the fine-tuned models consistently align with the collective expert agreement (the mode). In clinical practice, individual experts naturally exhibit inter-rater variance—sometimes assigning a score of 1 instead of 0 for ambiguous behaviors due to subconscious empathy associated with venipuncture. Because the PA-VLLF is trained on the consensus mode, it effectively filters out these individual subjective fluctuations, providing a highly standardized assessment baseline. A similar trend of strict model objectivity vs. human empathy is also observed across individual dimensions in Figures 4a–e.

### 4.4 Effect of prompt engineering

A zero-shot chain-of-thought prompt engineering for quantitative pain assessment improved the performance of PA-VLLF (Supplementary prompt engineering). The chain of thought that we designed contains three steps: (1) guide the model to assess the pain level of a subject step by step according to the FLACC scale in five dimensions; (2) summarize the scores; (3) convert the total score into a pain level.

**Figure 5:**
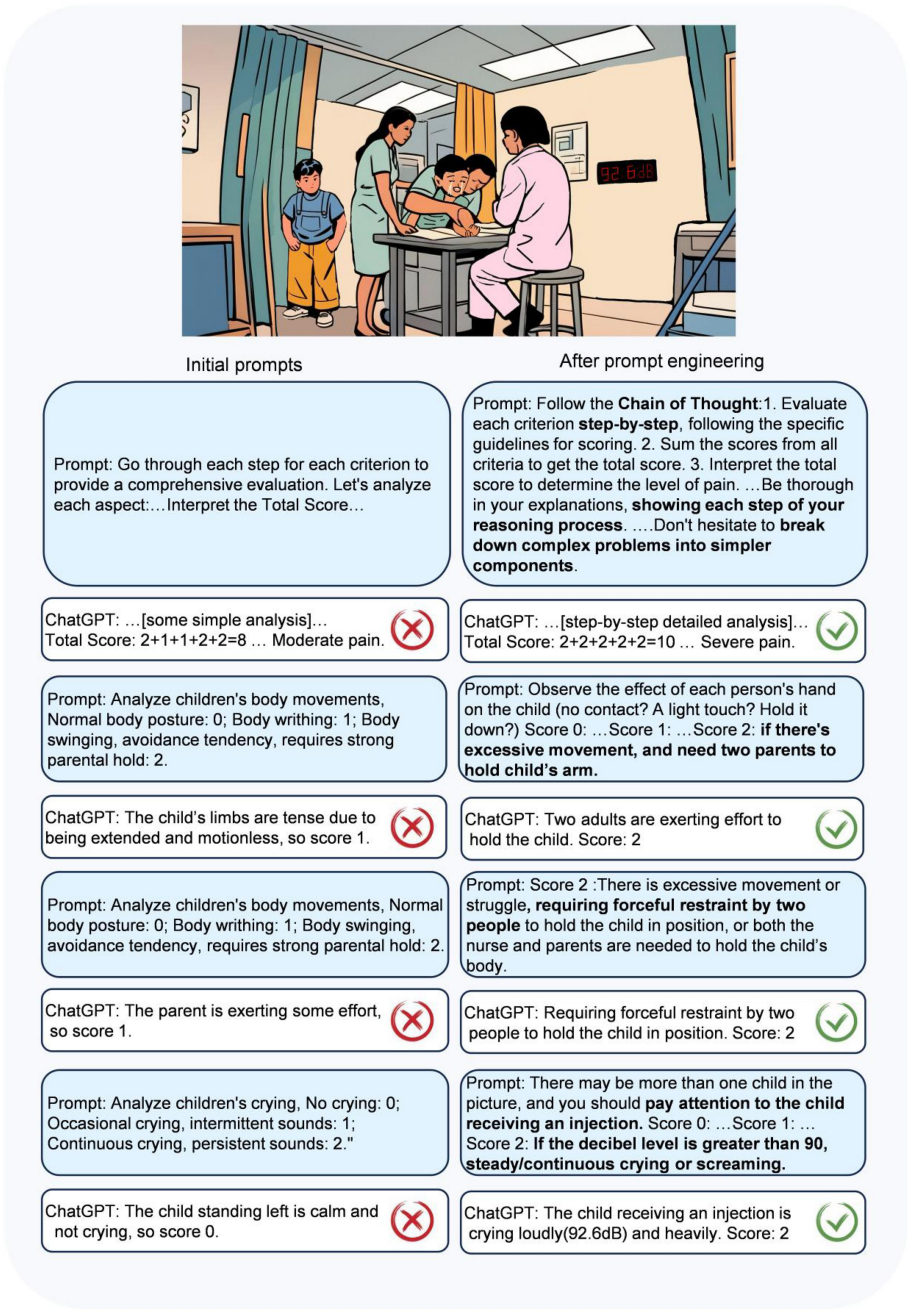
Zero-shot chain-of-thought prompt engineering. (The clinical scene depicted is an AI-generated illustration based on real clinical video frames to protect the privacy of patients and medical staff).

Figure 5 provides a concrete example of prompt engineering. Based on the initial prompt without engineering, the child's pain score in the image was calculated as 2 + 1 + 1 + 2 + 2, totaling 8 points, indicating moderate pain. In the leg and activity dimensions, the initial prompt led ChatGPT to mistakenly score the child's limbs as tense (1 point each) because they were extended and motionless. After engineering, PA-VLLF demonstrated advanced visual reasoning by recognizing that two adults were forcefully restraining the child's arms and body, leading to an adjusted score of 2 points each. Additionally, the initial prompt caused ChatGPT to incorrectly assess a bystanding child. After prompt engineering, the model correctly identified that only the child receiving the venipuncture should be scored.

Furthermore, the engineered prompt seamlessly integrated the pre-annotated Crying baseline (provided as contextual input, reflecting an acoustic state of 92.6 dB) with the model's independent visual assessments. As a result, the final scores were revised as 2 + 2 + 2 + 2 + 2, totaling 10, indicating severe pain, which is consistent with the ground truth provided by pain management experts.

### 4.5 Effect of fine-tuning

**Figure 6:**
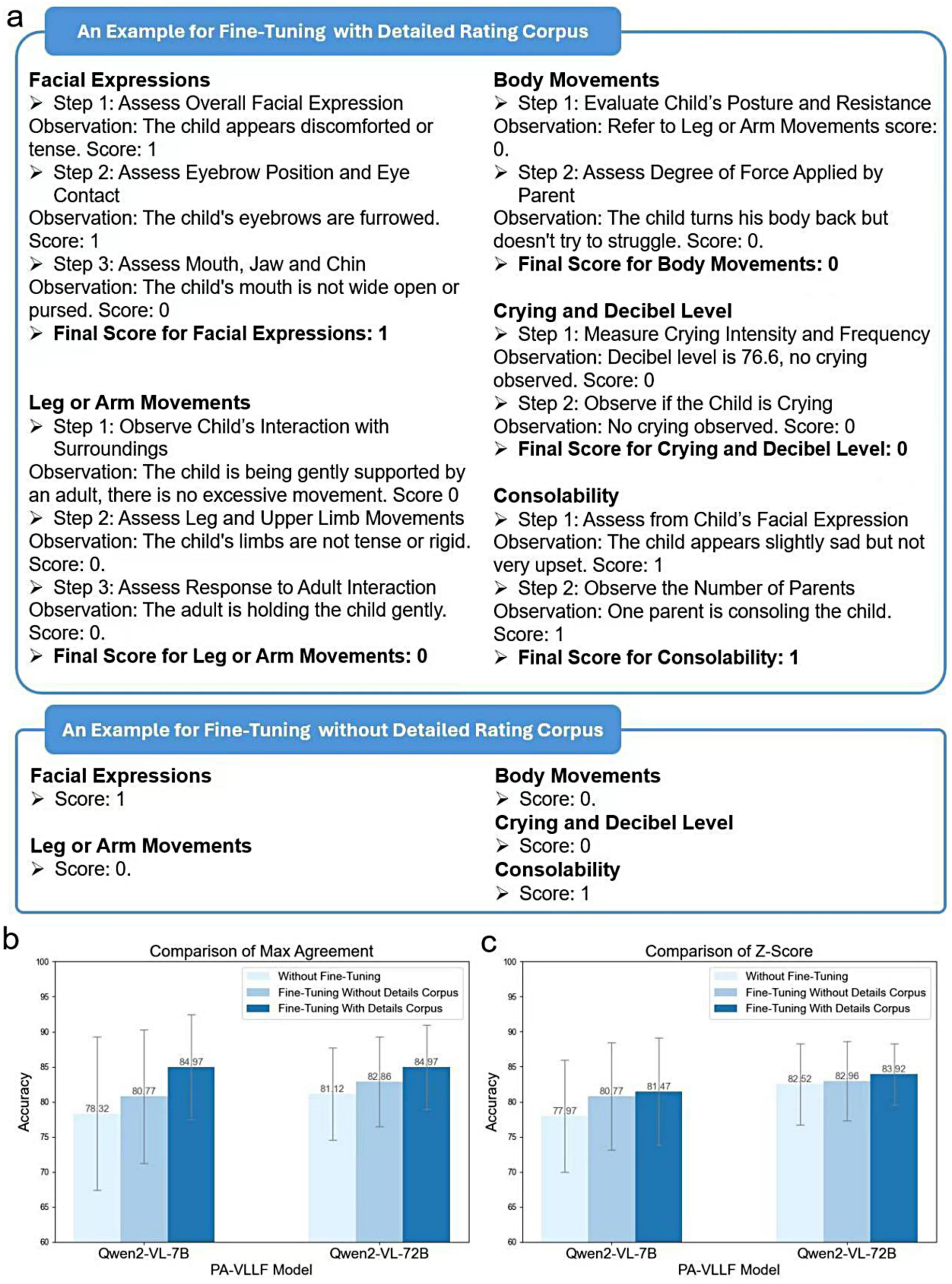
Comparison of fine-tuning. **(a)** An example of finetuning with and without Detailed Rating Corpus. **(b,c)** Comparison between Qwen2-VL-7B and Qwen2-VL-72B across Without Fine-Tuning, Fine-Tuning Without Details Corpus, and Fine-Tuning With Details Corpus on Max Agreement (Z-Score) metrics.

Fine-tuning with a detailed rating corpus improved the performance of PA-VLLF (Figure 6a). In the Max Agreement metric (Figure 6b), the fine-tuned 72B and 7B models achieved accuracies of 84.97%, exceeding their base versions by 3.85% and 6.65%, respectively. A similar trend was observed in the Z Score metric (Figure 6c), where the fine-tuned 72B and 7B models achieved 83.92% and 81.47%, surpassing their base versions by 1.4% and 3.5%, respectively.

Fine-tuning with a detailed rating corpus for each score from a pain management expert yields better pain assessment performance than that without a detailed rating corpus. We fine-tuned PA-VLLF-Qwen2-VL using the rating corpus with and without a detailed rating corpus. PA-VLLF-Qwen2-VL-7B and PA-VLLF-Qwen2-VL-72B with a detailed rating corpus outperform without a detailed rating corpus by 4.20% and 2.11%, respectively (Figures 6b,c).

## 5 Discussion

The major challenge in clinical pain management has been developing an effective tool for rapid, standardized and accurate pain assessment to ensure optimal pain treatment. PA-VLLF takes a significant step in this direction. Using representative keyframes extracted from two surveillance cameras, PA-VLLF achieves objective and expert-level pain assessment, without disrupting routine clinical workflows. Specifically, PA-VLLF demonstrates an expert-level precision that is comparable to senior pain management experts across overall pain levels and various key dimensions (e.g., facial expression, legs, and activity). In addition, PA-VLLF demonstrates a superior discriminative capacity in resolving ambiguous scores, effectively decreasing the influence of human subjectivity driven by empathy. In clinical deployment, therefore, the PA-VLLF system is designed to serve as an intelligent assistant for non-expert raters (e.g., trainee nurses or parents), rather than replacing senior clinicians. By providing an consistent, expert-level baseline, it helps reduce the subjective variance typical of less experienced staff and standardizes pain management across varying expertise levels. Furthermore, we wish to emphasize that the ultimate goal of developing the PA-VLLF is not to fully replace the nuanced judgment and empathy of professional healthcare providers. Rather, it is designed to serve as a highly efficient clinical decision-support tool. By providing rapid, consistent, and standardized baseline assessments, the PA-VLLF assists clinical staff in optimizing workflows, mitigating individual rater variance, and ultimately improving the efficiency of timely pain management interventions in busy real-world clinical settings.

The PA-VLLF training dataset was derived from clinical venipuncture for blood collection scenarios, enabling PA-VLLF to excel in assessing acute pain induced by procedural interventions in children. As a proof-of-concept, it holds significant promise for future adaptation to evaluate pain in pediatric patients undergoing various invasive procedures, ranging from finger and heel pricks, immunizations, and dressing changes to more intensive interventions such as lumbar punctures and bone marrow biopsies. Furthermore, PA-VLLF can aid in postoperative pain assessment, offering valuable information for the precise administration of postoperative analgesics.

Our data set originated in real-world clinical scenarios, characterized by significant diversity and unpredictability. This presented a major challenge for any machine vision algorithm to achieve consistent pain assessment. First, patients' pain responses varied widely; e.g., some cried with tightly closed eyes, while others shed tears with eyes open. Younger patients, with weaker arm strength, expressed pain more through body movements. Some patients clenched their fists, while others retracted their arms. Second, in practical scenarios, patients were sometimes partially obscured by masks, medical equipment, medical staff, or accompanying family members. Parents or relatives might have unintentionally blocked the view of the camera. A further challenge was how to distinguish subtle interactions, such as differentiating between a parent firmly holding a patient's arm to prevent movement and gently supporting it. Lastly, the real-world scenarios did not limit the number of children in view. If non-patients were captured, a machine vision system might incorrectly assess them. Therefore, non-contact pain assessment from the surveillance video frames we collected is a particularly difficult machine vision problem, presenting all of the traditional hard computer vision challenges: variability in imaging conditions, in subject pose, size, and orientation, occlusions, clutter, and distractors. These nuisance factors make the challenge of how to infer pain level from a broad range of individual overt behaviors especially difficult. This prompted us to turn to the latest generation of vision-language AI systems to approach this task.

Before detailing the specific visual boundaries and error patterns of the model, it is essential to contextualize the evaluation metrics regarding the Crying dimension. According to the FLACC scale, calculating a valid composite score (0–10) strictly requires inputs from all five dimensions. However, scoring Crying fundamentally relies on acoustic signals. Because our dataset comprises video recordings from real-world pediatric clinical settings, the inherent complex environmental noise precluded the capture of clean, isolated audio necessary for reliable crying analysis. Given these acoustic limitations, predicting this specific auditory dimension purely from visual inputs is scientifically unfounded. To maintain rigorous evaluation standards and fulfill the mathematical necessity of computing the composite score, the exact Crying score was therefore provided as a known contextual variable to both the PA-VLLF model and the human expert raters. Therefore, the near-perfect accuracy on this specific metric functions as a controlled baseline shared by both groups, rather than an independent predictive achievement of the model. Consequently, the true visual discriminative capacity of the PA-VLLF system—and its comparability to expert clinicians—is most accurately reflected in its robust performance across the four purely visual dimensions (Face, Legs, Activity, and Consolability).

While the PA-VLLF demonstrates significant advantages in addressing these real-world complexities, an in-depth error analysis of the misclassifications (Figure 4) and inter-expert agreement (Figure 3) reveals its current clinical boundaries. First, regarding the Leg Movement dimension, we observed notably lower inter-expert agreement (Figure 3) and significant AI overestimation. As explicitly shown in Figure 4b, ChatGPT-4o frequently misclassified a ground-truth score of 0 (Normal) as 1 or 2 (53 and 19 instances, respectively). Clinically, this “absent-as-severe” error occurs because anticipatory anxiety and fear often manifest as intense kicking or restlessness even before actual painful stimuli occur. Distinguishing between pain-induced reflex withdrawal and fear-induced squirming introduces high subjective variance among human experts, inherently causing vision models to over-predict pain based solely on excessive movement. Second, critical underestimations—such as downgrading Moderate or Severe pain to Mild—primarily occurred in extreme occlusion scenarios. For example, in the overall Pain Level assessment (Figure 4f), the models occasionally underestimated Moderate pain as Mild (e.g., 10 instances by Qwen2-VL-finetune). When a severely crying child completely buries their face into a parent's chest, the model is deprived of critical visual features (such as facial expressions), inevitably leading to this underestimation. Furthermore, differentiating the subtle semantics of physical touch—such as a parent firmly restraining an arm vs. gently supporting it—occasionally caused the model to misinterpret the “Consolability” dimension. Identifying these specific failure patterns provides clear directions for future algorithmic refinement.

Beyond refining the algorithm to address these specific visual boundaries, expanding the framework's adaptability to various clinical standards is another crucial advantage of our approach. The gold standard for pain assessment is self-reporting. However, for children who cannot accurately verbalize their pain, age-appropriate behavioral assessment tools are recommended. The FLACC scale, which serves as the foundational framework for PA-VLLF training, was specifically designed for populations unable to self-report pain and remains the most widely used tool in clinical practice. As it relies on observers to rate pain levels based on standardized criteria, it is well suited for training AI models. For nonverbal patients, additional observer-rated scales include the revised Neonatal Facial Coding System (NFCS-R) and the Observational Visual Analog Scale (obs VAS). NFCS-R provides a standardized method for quantifying neonatal pain intensity through facial expression analysis (35, 36), while obs VAS requires healthcare providers to mark pain intensity on a scale based on observed behaviors and physiological indicators (37). The large language model (LLM) framework used in this study can be adapted to other pain assessment scales. By clearly defining prompts, this approach facilitates the development of AI models as intelligent assistants for clinical observer-rated tools, demonstrating broad applicability and significant potential for clinical implementation.

Furthermore, while the current proof-of-concept study demonstrates the potential of the VLLF model through retrospective analysis of representative frames from clinical videos, deploying and validating the trained model in actual, real-time clinical scenarios remains a critical next step for our future research.

## 6 Limitations

Despite the promising performance of PA-VLLF in pediatric pain assessment, several limitations should be acknowledged.

Sample Size and Intra-Class Correlation: While our *a priori* power analysis indicated that 286 keyframes provide adequate statistical power for 4-class discrimination, the patient-level cohort (*n* = 104) remains limited. Extracting multiple keyframes from the same patient inherently introduces biological within-patient clustering. From a biostatistical perspective, this intra-class correlation reduces the effective patient-level sample size compared to the absolute image count, potentially slightly overestimating our initial power calculation.

Static Evaluation of Dynamic Behaviors: Evaluating temporal dimensions—such as Activity and Consolability in the FLACC scale—from static keyframes deviates from continuous clinical observation. Although these representative frames successfully captured peak pain expressions during venipuncture, the absence of continuous spatiotemporal behavioral tracking restricts the absolute completeness of the assessment.

Human-in-the-Loop Crying Assessment: The current framework requires manual pre-annotation for the Crying dimension. Complex environmental noise in real-world pediatric settings currently precludes reliable automated audio detection. Because calculating the composite 0–10 FLACC score mathematically mandates all five dimensions, this pre-annotated score was provided as a controlled baseline. Furthermore, due to the rapid deprecation of the original GPT-4.0 API used in our experiments, retrospectively forcing audio-visual predictions using mismatched, newer models would compromise internal methodological consistency. Therefore, the system's true discriminative capability is most accurately reflected in its performance on the four purely visual dimensions (Face, Legs, Activity, and Consolability), where it may help reduce the manual burden associated with repetitive visual pain assessment.

Generalizability and External Validation: The dataset exhibits a natural class imbalance (e.g., 68.53% “no pain”), reflecting true clinical distributions but potentially influencing model sensitivity across minor categories. Additionally, lacking a multi-center external validation cohort limits proven generalizability across diverse demographic populations. However, because our data derives from 6,240 min of unconstrained, continuous clinical surveillance rather than controlled laboratory environments, it intrinsically captures high real-world heterogeneity (e.g., lighting variations, dynamic medical occlusions), partially mitigating risks of severe overfitting.

Future Directions: Subsequent research will focus on three priorities: (1) prospective multicenter validation using larger, demographically diverse pediatric cohorts; (2) incorporation of temporal video-processing frameworks to capture dynamic behavioral trajectories; and (3) development of noise-resistant, multimodal audio-visual architectures to achieve fully automated evaluation. Together, these advances will further strengthen the generalizability and scalability of the PA-VLLF system.

## 7 Conclusion

In conclusion, PA-VLLF provides a proof-of-concept that vision-language large models can successfully internalize and consistently apply expert-derived clinical reasoning for FLACC-based pediatric pain assessment. By serving as a standardized consensus-based evaluation framework, it may support more consistent pain assessment and reduce the burden of repetitive visual evaluations. While external validation remains necessary, these findings highlight the potential of vision-language AI to assist pediatric pain assessment in real-world clinical settings.

## Data Availability

The original contributions presented in the study are included in the article/Supplementary Material, further inquiries can be directed to the corresponding author.
