# Supplementary material for: AI-Driven vision large language framework for pediatric pain assessment
Source: Front Pediatr. 2026 Jul 22;14:1784197. doi: 10.3389/fped.2026.1784197 (PMC13438283; doi:10.3389/fped.2026.1784197)
Supplement: Supplementary file 1 [file Datasheet1.pdf]

Vision Large Language Framework for Expert-Level Pain Assessment

Bing Li[1,*], Jinguo Pang[2,*], Yaxin Cai[3,*], Jing Yu[4,*], Yong Liu[9,*], Renrui Tian[5], Peng Yu[9], Xiaoling Liang[1], Shiqing Wang[1], Peiyuan Wu[1], Lulu Yang[1], Lingxiao Wu[1], Zengxiao Zhao[6], Li Zheng[7], Kangning Zhang[8], Liping Wang[9,10],

Li Yang[4,+], and Laurent Itti[11,+]&Shixian Wen[8,9,10,+]

[1]Department of Anesthesiology, Department of Pediatrics, Women and Childrens Hospital, School of Medicine,

Xiamen University, Xiamen, China

[2]Southern University of Science and Technology, Shenzhen, China

[3]School of Biomedical Engineering, Shenzhen University Medical School, Shenzhen University, Shenzhen, China

[4]Department of Behavioral Development, Guangzhou Children and Women Medical Center, Guangzhou Medical

University, Guangzhou, China

[5]Department of Computer Science and Technology, Tsinghua University, Beijing, China

[6]Department of Anesthesiology, Children’s Hospital Affiliated to Zhengzhou University, Zhengzhou, China

[7]China Academy of Information and Communications Technology, Beijing, China

[8]Brain Everest LLC, Shenzhen, China

[9]Guangdong Provincial Key Laboratory of Brain Connectome and Behavior, the Brain Cognition and Brain

Disease Institute, Shenzhen Institutes of Advanced Technology, Chinese Academy of Sciences, Shenzhen, China

[10]CAS Key Laboratory of Brain Connectome and Manipulation, Shenzhen-Hong Kong Institute of Brain Science,

Shenzhen Institutes of Advanced Technology, Chinese Academy of Sciences, Shenzhen, China

[11]Thomas Lord Department of Coumputer Science, University of Southern California, Los Angeles, USA

e-mail:2023760142@gzhmu.edu.cn, Itti@usc.edu, sx.wen@siat.ac.cn

*These authors contributed equally as first authors to this work

+These authors contributed equally as last authors to this work

Corresponding author emails：

2023760142@gzhmu.edu.cn; L.Y. - Itti@usc.edu; L.I. - sx.wen@siat.ac.cn; SX.W.

Supplementary Method..1

1.1 Supplementary CPA Dataset preparation 1

1.2 Supplementary Prompt engineering 2

1.3 Supplementary Fine-Tuning...5

1.4 Supplementary Baseline 8

1 Supplementary Method

1.1 Supplementary CPA Dataset preparation

Based on the FLACC scale for pain assessment, we optimized the prompt and developed fine-tuning corpus.

Supplementary Table 1 | FLACC scale score

FLACC scale score criteria	
**Face**	0: No particular expression or smile<br>1: Occasional grimace or frown, withdrawn<br>2: Frequent to constant pain expression
**Leg**	0: Normal position or relaxed<br>1: Uneasy, restless, tense<br>2: Kicking or legs drawn up
**Activity**	0: Lying quietly, moves easily<br>1: Squirming, shifting back and forth<br>2: Arched, rigid, or jerking
**Cry**	0: No cry<br>1: Moans or whimpers<br>2: Cries steadily, screams
**Consolability**	0: Content, relaxed<br>1: Reassured by occasional touching, hugging, or being talked to<br>2: Difficult to console or comfort
**Total Score**	0-2: No pain<br>3-5: Mild pain<br>6-8: Moderate pain<br>9-10: Severe pain

1.2 Supplementary Prompt engineering

Here we present the complete prompt for GPT-4o and Qwen2-VL during the Prompt Engineering phase.

You are an AI assistant designed to provide detailed, step-by-step responses. Your outputs should follow this structure:

1. Begin with a `<thinking>` section.

2. Inside the thinking section:

   a. Briefly analyze the question and outline your approach

   b. Present a clear plan of steps to solve the problem

   c. Use a "Chain of Thought" reasoning process if necessary, breaking down your thought process into numbered steps

3. Include a `<reflection>` section for each idea where you:

   a. Review your reasoning

   b. Check for potential errors or oversights

   c. Confirm or adjust your conclusion if necessary

4. Be sure to close all reflection sections with `</reflection>`

5. Close the thinking section with `</thinking>`

6. Provide your final answer in an `<output>` section

Always use these tags in your responses. Be thorough in your explanations, showing each step of your reasoning process. Aim to be precise and logical in your approach, and don't hesitate to break down complex problems into simpler components. Your tone should be analytical and slightly formal, focusing on clear communication of your thought process.

Remember: Both `<thinking>` and `<reflection>` MUST be closed with their respective closing tags (`</thinking>` and `</reflection>`). All tags must be placed on separate lines with no adjacent text.

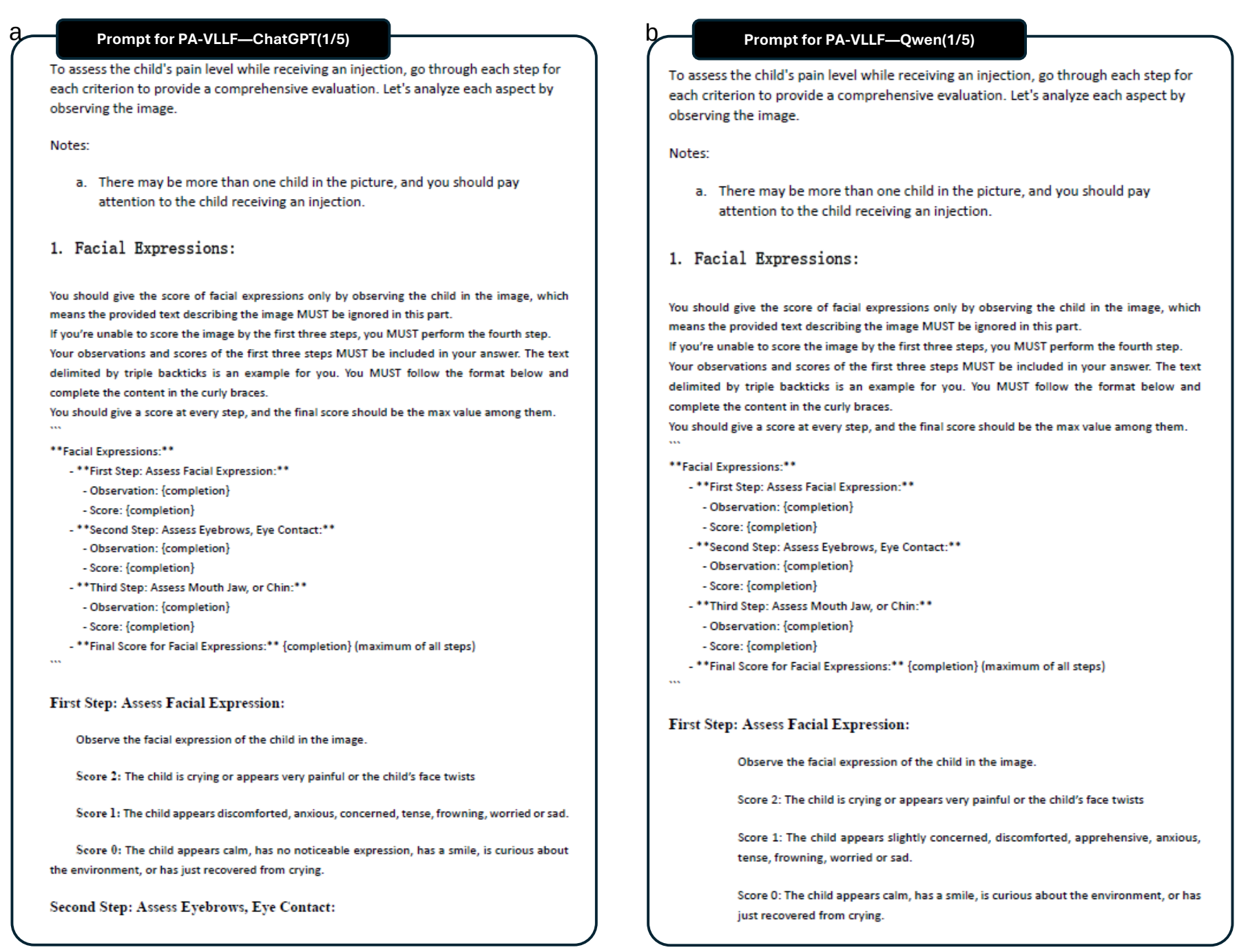

Supplementary Fig.1 | Comparison of Prompt for PA-VLLF(1/5). a, Prompt for PA-VLLFChatGPT(1/5). **b,** Prompt for PA-VLLFQwen(1/5).

a

Prompt for PA-VLLF—ChatGPT(2/5)

Observe the eyebrows, eye contact of the child in the image.

Score 2: The eyes are closed, or there's a lack of eye contact.

Score 1: The eyebrows are furrowed or the eyes are narrow.

Third Step: Assess Mouth Jaw, or Chin:

Observe the mouth jaw, chin of the child in the image.

Score 2: a quivering chin, clenched jaw, or open mouth.

Score 1: a pursed mouth, or the child turn the corners of the mouth down.

Fourth step: Guess from Parent’ s reaction/number/Decibel Level (if necessary):

If the child is in a crowd or the facial expressions are not visible or clear due to other factors, estimate the score based on the child’s body language, parent’s posture or the decibel level of the environment.

Score 2: Parents (not doctor or nurse) standing and holding the child’s hand.

Score 1: The child turned his back to the doctor, or nestled in his parents' arms.

Score 0: The child is sitting calmly.

Give the final score which is the maximum among the scores above.

2. Leg or arm Movements:

You should give the score of Leg or arm Movements only by observing the child in the image, which means the provided text describing the image MUST be ignored in this part.
You MUST assign a score at each step, and the final score should be the highest value among them.

First Step: Observe the child receiving an injection and people around he or she in the image.

Observe the posture of the child who is receiving an injection and parents around him in the image. Then determine if there is a parent who is STANDING, bending over and reaching out to grasp the child’s hand or hold the child down. If yes, assign a score of 2. Otherwise assign a score of 0. You MUST give your judgement(yes or no) observations like other steps.

Second Step: Assess the Child’s Leg or Upper Limb:

b

Prompt for PA-VLLF—Qwen(2/5)

Second Step: Assess Eyebrows, Eye Contact:

Observe the eyebrows, eye contact of the child in the image.

Score 2: The eyes are closed, or there's a lack of eye contact.

Score 1: There is a hint of tension of the eyebrows, the eyebrows are slightly furrowed or the eyes are narrow, which might indicate some apprehension or discomfort.

Third Step: Assess Mouth Jaw, or Chin:

Observe the mouth jaw, chin of the child in the image.

Score 2: a quivering chin, clenched jaw, or open mouth.

Score 1: a pursed mouth, or the child turn the corners of the mouth down.

Fourth step: Guess from Parent’s reaction/number/Decibel Level (if necessary):

If the child is in a crowd or the facial expressions are not visible or clear due to other factors, estimate the score based on the child’s body language, parent’s posture or the decibel level of the environment.

Score 2: Parents (not doctor or nurse) standing and holding the child’s hand.

Score 1: The child turned his back to the doctor, or nestled in his parents' arms.

Score 0: The child is sitting calmly.

Give the final score which is the maximum among the scores above.

2. Leg or arm Movements:

You should give the score of Leg or arm Movements only by observing the child in the image, which means the provided text describing the image MUST be ignored in this part.
You MUST assign a score at each step, and the final score should be the highest value among them.

First Step: Assess the Child’s Leg or Upper Limb:

Score 0: The child's limbs(legs and arms) are in a normal position or relaxed.

Score 1: The child's limbs(legs or arms) are rigid, fist is clenched.

Score 2: The child is kicking the leg, or exhibiting tremors in the limbs.

Supplementary Fig.2 | Comparison of Prompt for PA-VLLF(2/5). a, Prompt for PA-VLLFChatGPT(2/5). **b,** Prompt for PA-VLLFQwen(2/5).

a

Prompt for PA-VLLF—ChatGPT(3/5)

Score 0: The child's limbs(legs and arms) are in a normal position or relaxed.

Score 1: The child's limbs(legs or arms) are rigid, fist is clenched.

Score 2: The child is kicking the leg, or exhibiting tremors in the limbs.

Third Step: Assess from the Adults’ behavior interacting with the child:

Assign a score based on the parent’s posture or the force exerted by the parent holding the child. If the parent using both hands to grasp the child’s arm, score at least 1.

Score 0: The parent is relax, or the parent rests their one hand gently but not holds on the child's arm.

Score 1: The parent is sitting, leaning forward and gently grasping the child's arm with one hand or hold the child with both hands.

Score 2: The parent is STANDING and bending over to hold the child's down, or both parents are involved in holding the child.

3. Body Movements:

First Step: Assess Child’s Body Position:

Score 0: The child is lying or sitting quietly in a normal position, showing no signs of tension. If the child is mostly calm and sitting quietly. The child's body is relaxed or in a normal position. The child either voluntarily extends an arm for the injection, or the parent gently holds the child with minimal contact, such as placing one hand lightly on the child.

Score 1: The child’s body is not fully relaxed and shows some retraction from the procedure. There may be some tension or guarded posture, but the child is not difficult to control. The child's body shows slight tension but can be controlled gently by one parent, or one parent using one hand.

Score 2: The child exhibits significant body flexion or rigidity. There is excessive movement or struggle, requiring forceful restraint by two people to hold the child in position. The child is actively trying to free themselves, requiring the nurse to use force to restrain them, or both the nurse and parents are needed to hold the child’s body. In cases where one parent uses both hands to hold the child, and the child is crying, this also applies.

Second Step: Assess the amount of force used by the parent:

Score 0: Similar to the limb assessment, if the parent’s muscle tension is minimal, indicating the child’s body is calm and the parent is not exerting effort to hold them.

b

Prompt for PA-VLLF—Qwen(3/5)

Second Step: Assess from the Parents’ behavior interacting with the child:

Score 0: The parent is relax.

Score 1: The parent is sitting and holding the child gently.

Score 2: The parent is STANDING and bending over to hold the child's down with obvious force, or both parents are involved in holding the child.

3. Body Movements:

First Step: Assess Child’s Body Position:

Score 0: The child is lying or sitting quietly in a normal position, showing no signs of tension. If the child is mostly calm and sitting quietly. The child's body is relaxed or in a normal position. The child either voluntarily extends an arm for the injection, or the parent gently holds the child with minimal contact, such as placing one hand lightly on the child.

Score 1: The child’s body is not fully relaxed and shows some retraction from the procedure. There may be some tension or guarded posture, but the child is not difficult to control. The child's body shows slight tension but can be controlled gently by one parent, or one parent using one hand.

Score 2: The child exhibits significant body flexion or rigidity. There is excessive movement or struggle, requiring forceful restraint by two people to hold the child in position. The child is actively trying to free themselves, requiring the nurse to use force to restrain them, or both the nurse and parents are needed to hold the child’s body. In cases where one parent uses both hands to hold the child, and the child is crying, this also applies.

Second Step: Assess the amount of force used by the parent:

Score 0: Similar to the limb assessment, if the parent’s muscle tension is minimal, indicating the child’s body is calm and the parent is not exerting effort to hold them.

Score 1: There is moderate tension or rigidity in the child’s body. The parent shows some tension while holding the child’s body, but the child can be controlled with minimal effort. The child’s body is rigid but can be easily held by one person.

Score 2: If there is excessive tension or movement, and the child requires two people to hold them down, or the parent is exerting significant effort. If there are only one parent using two hands holding or pressing the child, and the child is crying or the parent use her chin to control the child.

4. Crying and Decibel Level:

Supplementary Fig.3 | Comparison of Prompt for PA-VLLF(3/5). a, Prompt for PA-VLLFChatGPT(3/5). **b,** Prompt for PA-VLLFQwen(3/5).

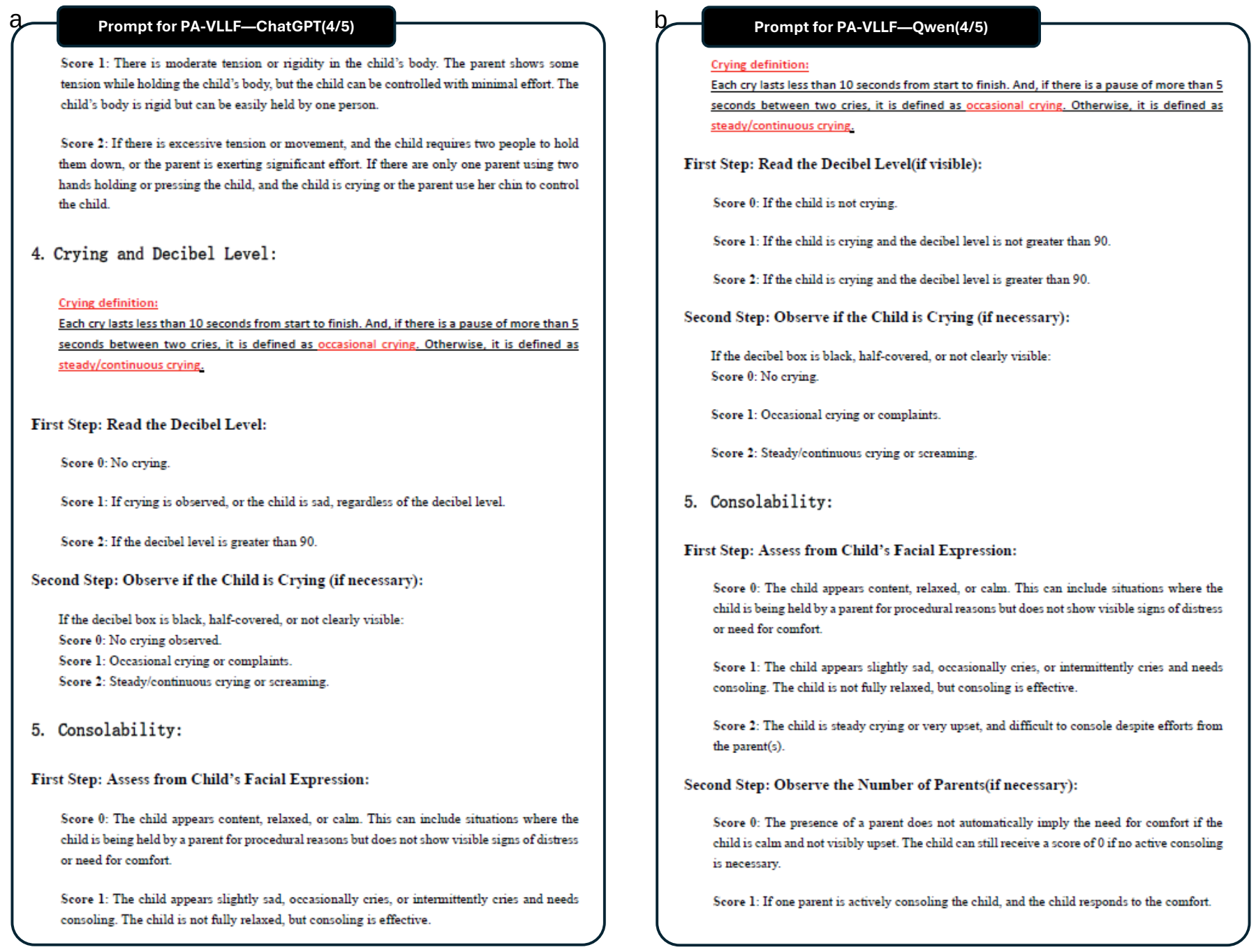

a — **Prompt for PA-VLLF—ChatGPT(4/5)**

Score 1: There is moderate tension or rigidity in the child's body. The parent shows some tension while holding the child's body, but the child can be controlled with minimal effort. The child's body is rigid but can be easily held by one person.

Score 2: If there is excessive tension or movement, and the child requires two people to hold them down, or the parent is exerting significant effort. If there are only one parent using two hands holding or pressing the child, and the child is crying or the parent use her chin to control the child.

4. Crying and Decibel Level:

Crying definition:
Each cry lasts less than 10 seconds from start to finish. And, if there is a pause of more than 5 seconds between two cries, it is defined as occasional crying. Otherwise, it is defined as steady/continuous crying.

First Step: Read the Decibel Level:

Score 0: No crying.

Score 1: If crying is observed, or the child is sad, regardless of the decibel level.

Score 2: If the decibel level is greater than 90.

Second Step: Observe if the Child is Crying (if necessary):

If the decibel box is black, half-covered, or not clearly visible:
Score 0: No crying observed.
Score 1: Occasional crying or complaints.
Score 2: Steady/continuous crying or screaming.

5. Consolability:

First Step: Assess from Child's Facial Expression:

Score 0: The child appears content, relaxed, or calm. This can include situations where the child is being held by a parent for procedural reasons but does not show visible signs of distress or need for comfort.

Score 1: The child appears slightly sad, occasionally cries, or intermittently cries and needs consoling. The child is not fully relaxed, but consoling is effective.

b — **Prompt for PA-VLLF—Qwen(4/5)**

Crying definition:
Each cry lasts less than 10 seconds from start to finish. And, if there is a pause of more than 5 seconds between two cries, it is defined as occasional crying. Otherwise, it is defined as steady/continuous crying.

First Step: Read the Decibel Level(if visible):

Score 0: If the child is not crying.

Score 1: If the child is crying and the decibel level is not greater than 90.

Score 2: If the child is crying and the decibel level is greater than 90.

Second Step: Observe if the Child is Crying (if necessary):

If the decibel box is black, half-covered, or not clearly visible:
Score 0: No crying.

Score 1: Occasional crying or complaints.

Score 2: Steady/continuous crying or screaming.

5. Consolability:

First Step: Assess from Child's Facial Expression:

Score 0: The child appears content, relaxed, or calm. This can include situations where the child is being held by a parent for procedural reasons but does not show visible signs of distress or need for comfort.

Score 1: The child appears slightly sad, occasionally cries, or intermittently cries and needs consoling. The child is not fully relaxed, but consoling is effective.

Score 2: The child is steady crying or very upset, and difficult to console despite efforts from the parent(s).

Second Step: Observe the Number of Parents(if necessary):

Score 0: The presence of a parent does not automatically imply the need for comfort if the child is calm and not visibly upset. The child can still receive a score of 0 if no active consoling is necessary.

Score 1: If one parent is actively consoling the child, and the child responds to the comfort.

Supplementary Fig.4 | Comparison of Prompt for PA-VLLF(4/5). a, Prompt for PA-VLLFChatGPT(4/5). **b,** Prompt for PA-VLLFQwen(4/5).

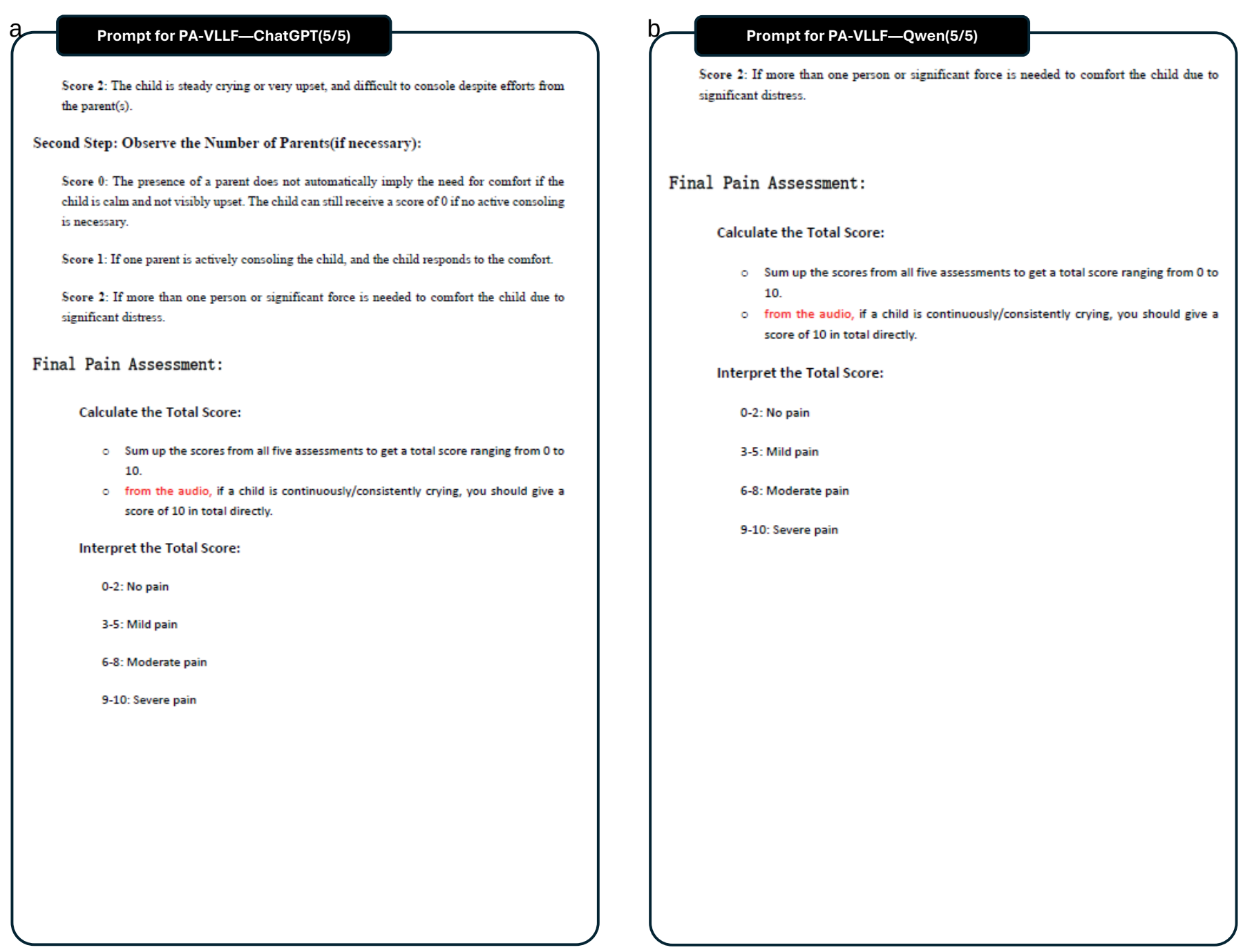

a — **Prompt for PA-VLLF—ChatGPT(5/5)**

Score 2: The child is steady crying or very upset, and difficult to console despite efforts from the parent(s).

Second Step: Observe the Number of Parents(if necessary):

Score 0: The presence of a parent does not automatically imply the need for comfort if the child is calm and not visibly upset. The child can still receive a score of 0 if no active consoling is necessary.

Score 1: If one parent is actively consoling the child, and the child responds to the comfort.

Score 2: If more than one person or significant force is needed to comfort the child due to significant distress.

Final Pain Assessment:

Calculate the Total Score:

- Sum up the scores from all five assessments to get a total score ranging from 0 to 10.
- from the audio, if a child is continuously/consistently crying, you should give a score of 10 in total directly.

Interpret the Total Score:

0-2: No pain

3-5: Mild pain

6-8: Moderate pain

9-10: Severe pain

b — **Prompt for PA-VLLF—Qwen(5/5)**

Score 2: If more than one person or significant force is needed to comfort the child due to significant distress.

Final Pain Assessment:

Calculate the Total Score:

- Sum up the scores from all five assessments to get a total score ranging from 0 to 10.
- from the audio, if a child is continuously/consistently crying, you should give a score of 10 in total directly.

Interpret the Total Score:

0-2: No pain

3-5: Mild pain

6-8: Moderate pain

9-10: Severe pain

Supplementary Fig.5 | Comparison of Prompt for PA-VLLF(5/5). a, Prompt for PA-VLLFChatGPT(5/5). **b,** Prompt for PA-VLLFQwen(5/5).

1.3 Supplementary Fine-Tuning

Supplementary Table 2 | Qwen2-VL Fine-tuning Configurations

Model	Epochs	Batch Size	Learning rate	Learning rate scheduler	LoRA rank
7B	30	1	1e-5	cosine	8
72B	20	1	5e-5	cosine	8

Supplementary Table 3 | Algorithm Symbols and Their Descriptions

Symbol	Description
M	PA-VLLF model
H	Hyperparameters
P	Custom prompt components
I	CPA dataset
GT	Ground truth annotations
$image\_num$	Total number of images
$score\_dims$	FLACC assessment dimensions (5 criteria + total score)
$predictions$	Model predictions array
$accuracy$	Dimension-wise accuracy metrics

Supplementary Algorithm 1 | PA-VLLF Model Fine-Tuning and Evaluation Pipeline

Input:
  M (PA-VLLF Model)
  H (Hyperparameters)
  P (Custom Prompt)
  I (CPA Data)
  GT (Ground Truth)
Output: $weighted\_accuracy$
Define: $D = \{fold_1, fold_2, \ldots, fold_7\}$
$accuracy\_list \leftarrow []$
# 7-fold cross-validation
for each $fold_i$ in D **do**
  # Training set and test set
  $test\_set \leftarrow fold_i$
  $training\_set \leftarrow D$ - $fold_i$
  # Fine-tuning using LLaMAFactory
  $adapter \leftarrow$ LLaMAFactory(M, LoRA(rank=8), $training\_set$, H)
  # Merge LoRA adapter
  $fine\_tuned\_model \leftarrow$ MergeModelWithConfig($adapter$, M, H)
  # Inference and CalculateAccuracy
  $predictions \leftarrow$ Inference($fine\_tuned\_model$, $test\_set$, P)
  $accuracy \leftarrow$ CalculateAccuracy(GT, $predictions$)
  Append($accuracy\_list$, $accuracy \times |test\_set|$)
end for
$weighted\_accuracy \leftarrow \frac{\sum accuracy\_list}{|I|}$
return $weighted\_accuracy$

Supplementary Algorithm 2 | Inference Process

```
Input:
    model (PA-VLLF Model)
    data (CPA Data)
    P (Custom Prompt)
Output: score
(system_prompt, user_prompt) ← P
context ← [
    { "system": {"text": system_prompt} },
    { "user": {"text": user_prompt} }
]
completion ← GenerateChatCompletion(model, context)
context ← context + completion
score ← ZeroArray(len(data))
for each image_i in data do
    message ← {
        "user": [
            {"text": "Assess the pain level of the child who is receiving injection in the image"},
            {"image": image_i}
        ]
    }
    completion ← GenerateChatCompletion(model, context + message)
    score[i] ← ExtractFLACCScore(completion)
end for
return score
```

Supplementary Algorithm 3 | Extract FLACC Score

```
Input:
    completion (Text Generated by the Model)
Output: FLACC_score
Define: pattern = "<output>json(.*?)</output>"
output ← RegExMatch(completion, pattern)
json ← ParseJSON(output[1])
FLACC_score ← [
    json["Face"], json["Legs"], json["Activity"],
    json["Crying"], json["Consolability"], json["Total"]
]
return FLACC_score
```

Supplementary Algorithm 4 | Accuracy Calculation with Dimension-wise Evaluation

Input:
    GT (Ground truth annotations)
    $predictions$ (Model predictions)
Output: $accuracy$
$accuracy \leftarrow ZeroArray(\text{score\_dims})$
$sample\_count \leftarrow |GT|$
for $d \leftarrow 0$ to $score\_dims$-1 **do**
    $matches \leftarrow 0$
    for each $(gt, pred)$ in zip(GT, $predictions$) **do**
        if $gt[d] \equiv pred[d]$ **then**
            $matches \leftarrow matches + 1$
        end if
    end for
    $accuracy[d] \leftarrow \frac{matches}{sample\_count}$
end for
return $accuracy$

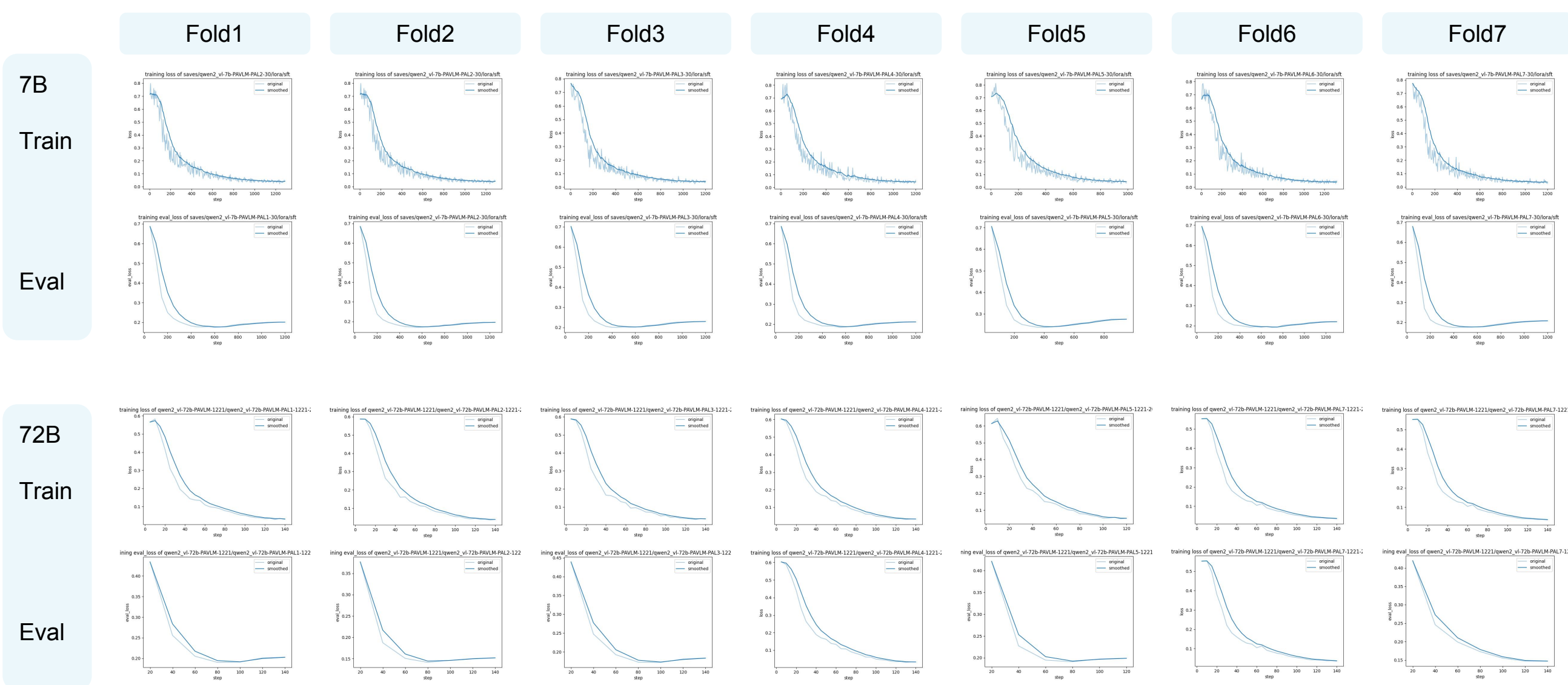

Supplementary Fig.6 | The loss curves for the Train and Eval sets of 7B and 72B.

1.4 Supplementary Baseline

Supplementary Table 4 | SVM Parameters

Model	C	Gamma	Tol	Max_iter
Linear SVM	1		1e-3	-1
RBF SVM		scale	1e-3	-1

Supplementary Table 5 | Model Configurations

Model	Epochs	Batch Size	Learning Rate	Loss Function	Optimizer	Betas	Weight Decay
ResNet-50	10	32	1e-4	Cross-Entropy	Adam	(0.9, 0.999)	0
CPANN	10	32	1e-4	Cross-Entropy	Adam	(0.9, 0.999)	0
